# A GWAS SNP for Schizophrenia Is Linked to the Internal MIR137 Promoter and Supports Differential Allele-Specific Expression

**DOI:** 10.1093/schbul/sbv144

**Published:** 2015-10-01

**Authors:** Alix Warburton, Gerome Breen, Vivien J. Bubb, John P. Quinn

**Affiliations:** ^1^Department of Molecular and Clinical Pharmacology, Institute of Translational Medicine, The University of Liverpool, Liverpool, UK;; ^2^MRC Social Genetic and Developmental Psychiatry Research Centre, Institute of Psychiatry, King’s College London, London, UK;; ^3^Biomedical Research Centre for Mental Health, South London and Maudsley NHS Foundation Trust and Institute of Psychiatry, National Institute for Health Research (NIHR), King’s College London, London, UK

**Keywords:** gene-environment interaction, linkage disequilibrium, microRNA-137, proxy SNP

## Abstract

Single nucleotide polymorphisms (SNPs) within the MIR137 gene locus have been shown to confer risk for schizophrenia through genome-wide association studies (GWAS). The expression levels of microRNA-137 (miR-137) and its validated gene targets have also been shown to be disrupted in several neuropsychiatric conditions, including schizophrenia. Regulation of miR-137 expression is thus imperative for normal neuronal functioning. We previously characterized an internal promoter domain within the MIR137 gene that contained a variable number tandem repeat (VNTR) polymorphism and could alter the in vitro levels of miR-137 in a stimulus-induced and allele-specific manner. We now demonstrate that haplotype tagging-SNP analysis linked the rs1625579 GWAS SNP for schizophrenia to this internal MIR137 promoter through a proxy SNP rs2660304 located at this domain. We postulated that the rs2660304 promoter SNP may act as predisposing factor for schizophrenia through altering the levels of miR-137 expression in a genotype-dependent manner. Reporter gene analysis of the internal MIR137 promoter containing the common VNTR variant demonstrated genotype-dependent differences in promoter activity with respect to rs2660304. In line with previous reports, the major allele of the rs2660304 proxy SNP, which has previously been linked with schizophrenia risk through genetic association, resulted in downregulation of reporter gene expression in a tissue culture model. The genetic influence of the rs2660304 proxy SNP on the transcriptional activity of the internal MIR137 promoter, and thus the levels of miR-137 expression, therefore offers a distinct regulatory mechanism to explain the functional significance of the rs1625579 GWAS SNP for schizophrenia risk.

## Introduction

MIR137 has been replicated as a strong schizophrenia candidate gene through genome-wide association.^1,2^ MIR137 encodes for microRNA-137 (miR-137), which has been identified as a modulator of processes implicated in schizophrenia pathogenesis including neurodevelopment, adult neurogenesis, synaptogenesis, and neural transmission.^3–6^ Other genome-wide association studies (GWAS) candidate genes for schizophrenia have also been validated as miR-137 targets,^1,3,7,8^ suggesting that dysregulation of the miR-137 pathway may be a key mechanism in neuropsychiatric dysfunction.

We recently characterized an internal promoter within the MIR137 gene locus, which we termed Imir137, and validated its potential to modulate the levels of miR-137 expression in vitro using a reporter gene system.^9^ The Imir137 promoter encompasses a variable number tandem repeat (VNTR) domain, which has previously been shown to modulate the processing and function of miR-137 in melanoma cell lines.^10^ We demonstrated the ability of this polymorphic domain to modify the transcriptional activity of the Imir137 promoter in an allele-specific and stimulus-inducible manner.^9^ Haplotype analysis of the MIR137 gene locus using genotype data from the HapMap CEU cohort showed that the MIR137 GWAS single nucleotide polymorphisms (SNPs) for schizophrenia were not in linkage disequilibrium (LD) with the MIR137 VNTR.^9^ In this communication we show that the rs1625579 GWAS SNP does, however, tag the rs2660304 SNP located at the identified Imir137 promoter. Both rs1625579 and the rs2660304 promoter SNP have been shown to modulate the levels of miR-137 expression.^11,12^ In this manuscript we propose a mechanism by which rs2660304 modulates miR-137 levels.

## Methods

### LD Analysis

SNP genotype data spanning chromosome 1: 98,075,522-98,711,836 corresponding to individuals from the CEPH trios of European descent was downloaded from the HapMap Genome Browser (http://hapmap.ncbi.nlm.nih.gov/), release #28. Haplotype tagging SNP analysis was performed using Haploview 4.1 (www.broad.mit.edu/mpg/haploview/) under the Linkage Format feature (Hardy-Weinberg *P* value cut-off, .001; minimum genotype cut-off, 75%; maximum number of Mendel errors, 1; minimum minor allele frequency, 0.01) and pair-wise tagging analysis performed (*r* ^2^ threshold, 0.8). Haplotype blocks were determined using 95% confidence intervals.^13^

### HaploReg

In order to annotate the potential regulatory effects of rs2660304 on transcription factor binding site motifs, the publically available HaploReg v3 software was employed (accessible at http://www.broadinstitute.org/mammals/haploreg/haploreg_v3.php). This calculates allele-specific changes in the log-odds (LOD) scores for position weight matrices (PWMs) of a regulatory motif based on TRANSFAC, JASPAR, and PBM databases.^14^

### Plasmid Construction

All MIR137 constructs used in this communication were cloned in the pGL3-Basic (pGL3B) luciferase reporter vector system (Promega). The Imir137 promoter construct containing the 4-copy variant of the MIR137 VNTR, termed Imir137(4), is described elsewhere.^9^ Imir137(4) fragments plus an additional 69bp of 5′ flanking sequence containing alternative alleles of the rs2660304 SNP were amplified using the following forward and reverse primers 5′-ATACCTCGAGACCCAAGAATACCCGTCA-3′ and 5′-ATACACGCGTTCATACCACCTAGAGTGGAC-3′ and cloned into the pGL3B vector at the XhoI and MluI restriction sites, respectively, indicated by the underlined sequences. The Imir137(4) constructs containing the major and minor alleles of the rs2660304 SNP were termed Imir137(4)+A and Imir137(4)+C, respectively.

### Cell Culture and Luciferase Reporter Gene Assays

The human-derived SH-SY5Y neuroblastoma cell line (*ATCC number CRL-2266*) was maintained as described previously.^15^ For luciferase assays, SH-SY5Y cells were seeded into 24-well plates at approximately 100 000 cells per well and transfected with 1 µg plasmid DNA and 10ng pMLuc2 (Novagen) (internal control for transfection efficiency) using TurboFect (Thermo Scientific). Transfected cells were processed 48 hours posttransfection using the Dual-Luciferase Reporter Assay System (Promega). Fold changes in firefly luciferase activity (normalized to renilla luciferase activity) supported by the Imir137 promoter domains over the pGL3B controls were calculated and significance determined using 2-tailed *t* tests. Significance was scored as follows **/##*P* < .01, ***/###*P* < .001. For each transfection, *n* = 4.

## Results

### Haplotype Structure of the MIR137 Gene

**Fig. 1.**
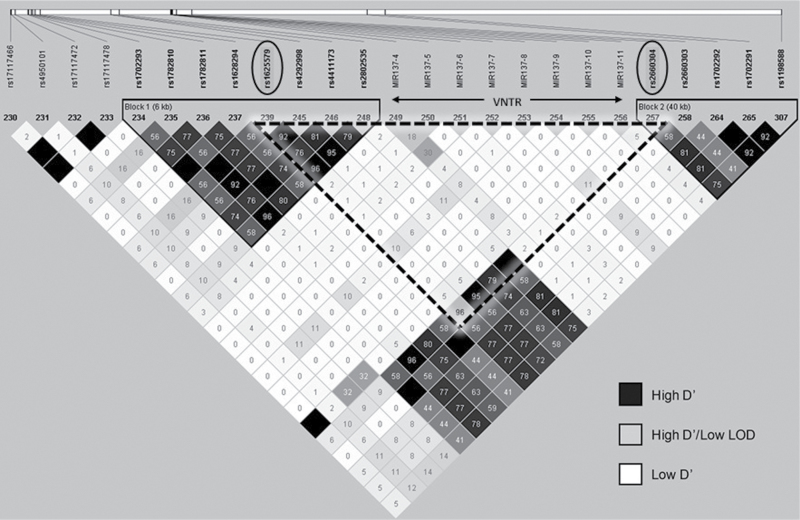
Linkage disequilibrium (LD) analysis of MIR137 gene locus. Haplotype block structure of the MIR137 gene based on squared correlation coefficient (*r* ^2^) values calculated from 89 individuals from the CEPH collection of the International HapMap Project using the Linkage Format feature in Haploview 4.2 (Hardy-Weinberg *P* value cut-off, .001; minimum genotype cut-off, 0.75; maximum number of Mendel errors, 1; minimum minor allele frequency, 0.01). Single nucleotide polymorphisms (SNPs) spanning chromosome 1: 98,075,522-98,711,836 were downloaded from the HapMap Genome Browser, release #28 (http://hapmap.ncbi.nlm.nih.gov/index.html.en). Haplotype blocks, represented by a solid black triangular border, were determined using 95% confidence intervals proposed by Gabriel et al^13^ which defined 2 haplotype blocks separated by the MIR137 variable number tandem repeat (VNTR) which is located within a recombination hot spot represented by white squares. Pair-wise tagging SNP analysis (*r* ^2^ > 0.8) revealed that the genome-wide association studies (GWAS) SNP rs1625579 and the MIR137 VNTR were not in LD. However, high LD (*r* ^2^ = 0.96) was observed between rs1625579 and the internal MIR137 promoter SNP rs2660304, demarcated by a dashed triangular border.

**Fig. 2.**
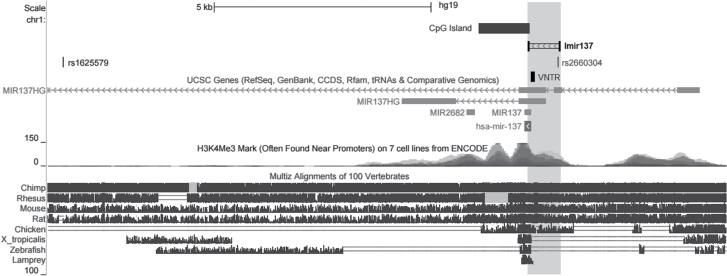
Internal MIR137 promoter. Schematic showing the location of rs2660304 within the internal MIR137 (Imir137) promoter and its relation to the rs1625579 genome-wide association studies (GWAS) single nucleotide polymorphism (SNP). Highlighted region represents that targeted by PCR primers (labeled Imir137) for cloning into the pGL3-Basic luciferase vector system. Multiz Alignments of 100 Vertebrates displays measurements of evolutionary conservation using PHAST Package. Pairwise alignments of each species to the human genome are shown with peak height indicating higher levels of overall conservation. Double lines indicate one or more bases in the gap region that do not align to the reference genome suggesting excessive evolutionary distance between species; pale shaded regions represent lack of sequence data between the aligning species. Image taken from the UCSC Genome Browser, hg19: chr1:98501896-98515504, accessed from http://genome.ucsc.edu/index.html.

The functional significance of the previously identified rs1625579 GWAS SNP for schizophrenia located within the third intron of the MIR137 gene remains elusive. Pair-wise tagging SNP analysis using genotype data from the HapMap CEU cohort revealed that the intronic GWAS SNP rs1625579 and a SNP rs2660304 situated 373bp upstream of the precursor (pre)-miR-137 were in strong LD (*r* ^2^ = 0.96), figure 1. As previously reported the MIR137 VNTR was not found to be in LD with the GWAS SNP nor tagged by any other markers within the same haplotype blocks.^9^ The rs2660304 proxy SNP therefore provided a distinct regulatory mechanism linking the GWAS variant to the Imir137 promoter, which can modify the levels of miR-137 based on VNTR copy number.^9^ Figure 2 illustrates the position of rs2660304 in relation to the Imir137 promoter and the rs1625579 GWAS SNP. Evolutionary conservation over the MIR137 gene locus across multiple vertebrates is also depicted indicating strong mammalian conservation not only in exonic regions and the sequence encompassing MIR137 but also in intronic and intergenic regions, as determined by the height of peaks of sequence homology, figure 2.

### The Major Risk Allele of rs2660304 Downregulates Imir137 Promoter Activity

**Fig. 3.**
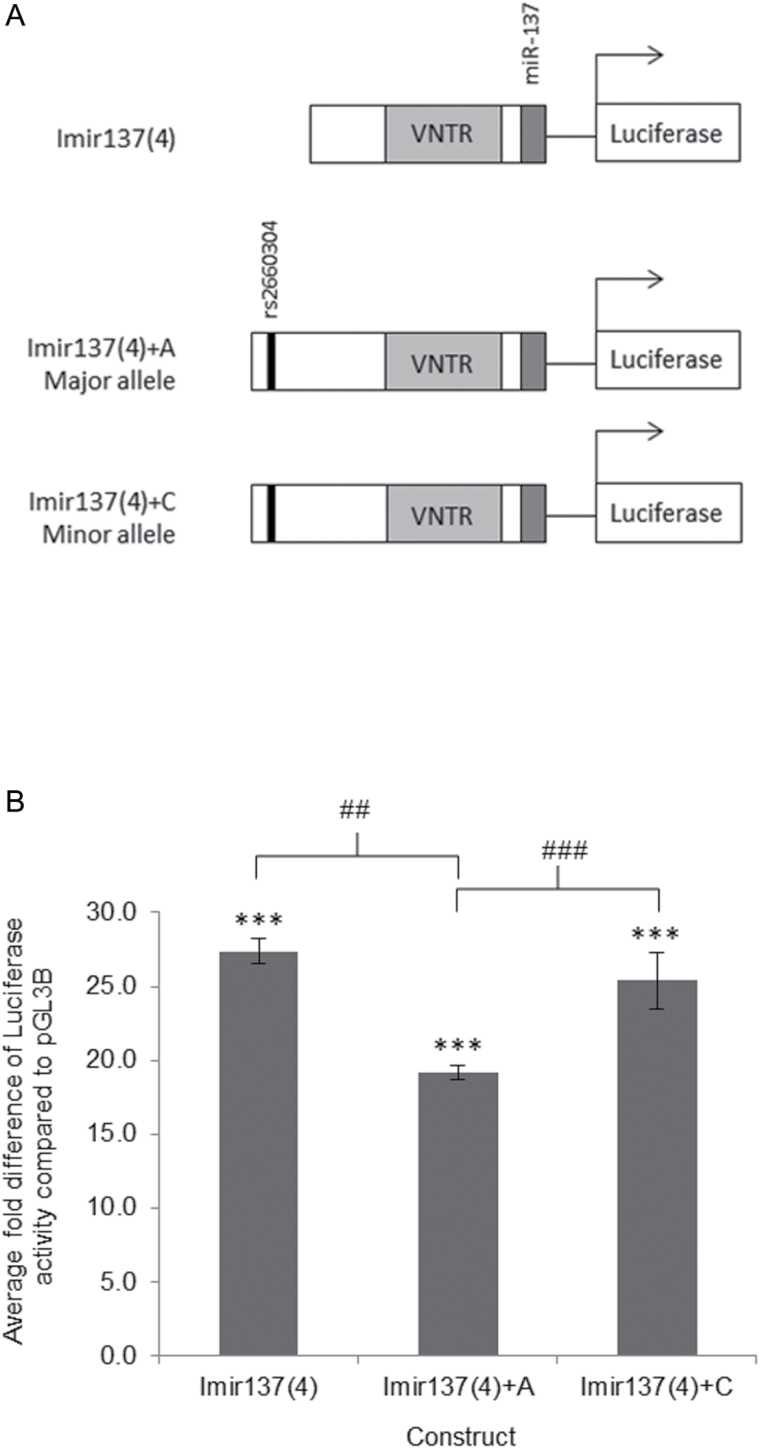
Functional analysis of the internal MIR137 promoter single nucleotide polymorphism (SNP) rs2660304. (A) Schematic representation of the internal MIR137 promoter (Imir137) constructs containing the 4-copy variable number tandem repeat (VNTR), plus or minus the rs2660304 SNP. The Imir137(4) construct lacking rs2660304 differs from the Imir137(4)+A (major allele) and Imir137(4)+C (minor allele) constructs by 69bp of sequence. (B) Luciferase activity supported by the Imir137 constructs in SH-SY5Y under basal conditions. *N* = 4. *Significant changes in luciferase activity over backbone control. ^#^Significant changes in luciferase activity between experimental conditions. ^##^ *P* < .01, ***/^###^ *P* < .001.

The functional significance of rs2660304 was addressed using reporter gene constructs containing the common 4-copy variant of the Imir137 promoter VNTR. These constructs differed from the Imir137(4) construct previously reported^9^ by an extension to include 69 nucleotides containing the rs2660304 SNP, 5′ of the original promoter sequences analyzed, and were termed Imir137(4)+A for the major allele and Imir137(4)+C for the minor allele (MAF; 0.168 in HapMap CEU), see figure 3A. Sequencing confirmed that these constructs varied only at the position of rs2660304. A significant difference in reporter gene activity was observed between the 2 alleles in SH-SY5Y cells (figure 3B, ###*P* < .001). The Imir137(4)+A construct containing the major allele of rs2660304 supported a 1.32-fold reduction (###*P* < .001) and 1.43-fold reduction (##*P* < .01) in reporter gene activity relative to the minor allele Imir137(4)+C and the shorter construct Imir137(4), respectively, figure 3B. This genotype-dependent reduction in promoter function was consistent with previous studies showing that the major allele of rs2660304 is associated with downregulation of miR-137 expression.^11,12^ There was no significant difference in luciferase activity between Imir137(4) and the minor allele containing Imir137(4)+C construct (figure 3B), supporting the conclusion that the difference in expression directed by the Imir137(4)+A and Imir137(4)+C constructs was a function of the SNP as the extended fragments were otherwise identical.

The potential regulatory function of the rs2660304 SNP on the Imir137 promoter was addressed using HaploReg v3, an online resource compiling information relating to epigenetic signatures, transcription factor binding sites, regulatory motifs and expression quantitative trait loci (eQTLs) relating to an expanded list of markers based on dbSNP-137.^14^ Using this web-based tool, the presence of the major risk allele for the rs2660304 proxy SNP correlated with an increased PWM score for the p53 transcription factor binding motif relative to the minor allele. Computational modeling of SNP-associated changes in PWM scores for a discovered transcription factor binding site motif, such as p53 in this case, highlight regions of potential perturbation in the regulation of gene expression through altering transcription factor binding and alternative splicing.

## Discussion

MIR137 has been identified as a gene that has a significant association with schizophrenia based on GWAS data.^1,2^ However the location of the associated SNPs within noncoding sequences suggests that the functional significance could be related to transcriptional or posttranscriptional regulation of the MIR137 gene. We have previously demonstrated the presence of a functional promoter in the region adjacent to the sequence of miR-137 itself and thus internal to the main precursor message.^9^ This promoter contained a VNTR, the genotype of which could mediate differential reporter gene expression. This VNTR has a large number of variants in the HapMap CEU population and is not in LD with the previously identified MIR137 GWAS SNPs,^9^ figure 1. However, haplotype analysis over the MIR137 locus showed strong LD (*r* ^2^ = 0.96) between the schizophrenia-associated SNP rs1625579 with a SNP rs2660304 within the identified Imir137 promoter (figures 1 and 2). Absence of LD between these markers and the MIR137 VNTR which is flanked by the 2 SNPs is reflective of its highly polymorphic nature and compatible with this repetitive element mutating through recombination, as indicated by white regions of low D’ on the LD plot shown in figure 1.

Due to the location of the rs1625579 GWAS SNP for schizophrenia within the first intron of the MIR137 gene, approximately 8.7kb upstream of pre-miR-137, we postulated that the rs2660304 promoter SNP located only 373bp upstream of pre-miR-137 may act as a proxy for the GWAS SNP, representing an alternative regulatory mechanism for disease predisposition through direct modulation of the Imir137 promoter. Superimposed on the potential action of the GWAS proxy SNP in regulating miR-137 expression via the internal promoter, the MIR137 VNTR could further modulate this regulatory domain as we have previously demonstrated.^9^ This was in part through the action of the transcription factor REST/NRSF (restrictive element-1 silencing transcription factor/neuron restrictive silencing factor). In addition, the MIR137 VNTR has previously been shown to affect the processing efficiency of miR-137 in melanoma cell lines, potentially through altering the secondary structure of the primary transcript in which it is located as determined by computational modelling.^10^ This would support a gene-environment interaction (G×E) through which VNTR genotype could act mechanistically to influence disease phenotype through altering the levels of miR-137 from the GWAS-linked internal promoter.

Analysis of the transcriptional potential of the promoter SNP using reporter gene constructs containing the common 4-copy variant of the MIR137 VNTR and the different alleles of rs2660304 showed allele-specific regulation in SH-SY5Y neuroblastoma cells. The construct containing the major allele of rs2660304 (Imir137(4)+A) demonstrated reduced activity relative to the Imir137(4)+C minor allele construct. This difference in reporter gene activity was specific to the rs2660304 SNP as expression levels supported by the Imir137(4)+C (minor allele) construct, whose sequence is identical to Imir137(4)+A other than at the SNP, did not significantly differ from the shorter parental Imir137(4) construct (figure 3). The major allele of the rs1625579 GWAS SNP is defined as the risk variant for schizophrenia,^1^ and has previously been associated with reduced miR-137 expression levels in post-mortem brains of homozygous individuals relative to carriers of the minor nonrisk allele.^11^ The significant reduction in reporter gene activity supported by the promoter construct containing the major allele of rs2660304, a SNP that is in LD with the rs1625579 GWAS SNP for schizophrenia (figure 1), is consistent with the above in vivo finding and implicates this promoter SNP and the Imir137 promoter VNTR as a potential mechanism driving differential miR-137 expression. Recently a study found a genetic correlation between rs2660304 and endogenous MIR137 expression in human fibroblast-derived neuronal cells, which again supported lower levels of MIR137 in association with the major risk allele.^12^ This study also addressed the transcriptional properties of this SNP but when directed by a heterologous minimal promoter in the pGL3 vector system and using a larger fragment encompassing both this SNP and the Imir137 promoter^12^; as such there could be confounding affects from having 2 transcriptional start sites in their construct. We have extended their analysis by demonstrating allele-specific expression of the rs2660304 SNP driven by its own internal promoter, Imir137,^9^ figure 3B. The role of rs2660304 in the pathophysiology of schizophrenia is further supported by a recent study that reported a nominal genetic association of this SNP with this neuropsychiatric condition,^16^ which strengthens its position as a proxy SNP for the rs1625579 schizophrenia variant, figure 1.

The rs1625579 GWAS SNP for schizophrenia has been reported to correlate with specific endophenotypes of the condition, with the major allele associating with cognitive deficits relating to working memory and executive function in individuals with negative symptoms; miR-137 expression levels; structural variations in the brain and earlier age of onset relative to carriers of the nonrisk allele.^11,17–19^ Other studies have failed to identify such genetic correlations,^20,21^ suggesting that the functional significance of rs1625579 may be reflective of additive interactions between genetic variants within the MIR137 gene locus, other susceptibility loci and/or pathways modulated by miR-137. This study was limited in that we did not address the synergistic effects of the rs2660304 proxy SNP with MIR137 VNTR copy number on Imir137 promoter function. Nor did we address the role of SNPs or INDELS within the MIR137 VNTR previously associated with modulating miR-137 expression levels in vitro.^4^ Variants within promoter VNTRs may act as clinical correlates of disease through G×E mechanisms as demonstrated by the rs25531 SNP within the long allele of the 5-HTT-linked polymorphic region (5-HTTLPR), whereby the presence of the minor allele of this variant confers clinical phenotypes normally associated with the short “risk” allele of 5-HTTLPR through modifying an AP2 transcription factor binding site.^22,23^ *In silico* analysis of the potential regulatory effects of the rs2660304 SNP on promoter function identified a compositional change in the binding motif for the transcriptional regulator p53, indicating an increase in binding potential relative to the minor allele. p53 plays a crucial role in neurological development^24^ and has been shown to be modulated in response to neuronal damage both in vitro and in vivo, reviewed in ref.^25^. Genetic association has also implicated p53 in the pathogenesis of schizophrenia,^26^ suggesting a potential regulatory network involving miR-137 and p53 as previously demonstrated in multiple myeloma cell lines.^27^

In summary, the data presented in this study provides a direct regulatory mechanism for the genetic correlation of the rs1625579 GWAS SNP with miR-137 expression levels through the action of the rs2660304 proxy SNP, which we demonstrated can modulate allele-specific expression driven from the Imir137 promoter using a reporter gene system. The role of the rs2660304 SNP in regulating expression from the Imir137 promoter is distinct from the action of the MIR137 VNTR, which also influences allele-specific and stimulus-inducible expression from this regulatory domain, due to absence of LD between these 2 polymorphisms. Strong LD exists between the GWAS SNP rs1625579 and rs2660304 within the Imir137 promoter suggesting that these SNPs may flank an associated haplotype that confers risk for schizophrenia, which may include rare high copy number variants of the VNTR such as the 12-copy variant previously demonstrated in our reporter gene assays to be functionally distinct from the common 4-copy variant. The importance of these variants in disease predisposition may only be apparent at the level of G×E; the cellular response to which can be dynamically shaped by cell-specific or stimulus-induced activation of transcriptional and epigenetic regulators such as REST/NRSF shown previously by our group to be one such factor operating at this complex disease locus.^9^

## Funding

Biotechnology and Biological Sciences Research Council (grant number: BB/F016905/1 to A.W., V.J.B., J.P.Q.).
